# The integrative process promoted by EMDR in dissociative disorders: neurobiological mechanisms, psychometric tools, and intervention efficacy on the psychological impact of the COVID-19 pandemic

**DOI:** 10.3389/fpsyg.2023.1164527

**Published:** 2023-09-01

**Authors:** Andrea Poli, Francesco Cappellini, Josephine Sala, Mario Miccoli

**Affiliations:** Department of Clinical and Experimental Medicine, University of Pisa, Pisa, Italy

**Keywords:** EMDR, dissociation, psychological trauma, structural dissociation, polyvagal theory, psychometrics, vagus nerve, integration

## Abstract

Dissociative disorders (DDs) are characterized by a discontinuity in the normal integration of consciousness, memory, identity, emotion, perception, bodily representation, motor control, and action. The life-threatening coronavirus disease 2019 (COVID-19) pandemic has been identified as a potentially traumatic event and may produce a wide range of mental health problems, such as depression, anxiety disorders, sleep disorders, and DD, stemming from pandemic-related events, such as sickness, isolation, losing loved ones, and fear for one's life. In our conceptual analysis, we introduce the contribution of the structural dissociation of personality (SDP) theory and polyvagal theory to the conceptualization of the COVID-19 pandemic-triggered DD and the importance of assessing perceived safety in DD through neurophysiologically informed psychometric tools. In addition, we analyzed the contribution of eye movement desensitization and reprocessing (EMDR) to the treatment of the COVID-19 pandemic-triggered DD and suggest possible neurobiological mechanisms of action of the EMDR. In particular, we propose that, through slow eye movements, the EMDR may promote an initial non-rapid-eye-movement sleep stage 1-like activity, a subsequent access to a slow-wave sleep activity, and an oxytocinergic neurotransmission that, in turn, may foster the functional coupling between paraventricular nucleus and both sympathetic and parasympathetic cardioinhibitory nuclei. Neurophysiologically informed psychometric tools for safety evaluation in DDs are discussed. Furthermore, clinical and public health implications are considered, combining the EMDR, SDP theory, and polyvagal conceptualizations in light of the potential dissociative symptomatology triggered by the COVID-19 pandemic.

## 1. Introduction

Dissociation is a complex, multifaceted phenomenon, defined as “a disruption of and/or discontinuity in the normal, subjective integration of one or more components of psychological functioning, including, but not limited to, identity, memory, consciousness, perception, and motor control” (Spiegel et al., 2011, p.826). This concept encompasses a wide range of somatoform symptoms (e.g., analgesia), as well as psychological symptoms, such as depersonalization, derealization, emotional numbness, and memory fragmentation (Spiegel and Cardeña, 1991; Waller et al., 1996; Holmes et al., 2005). Positive symptoms of dissociation include the involuntary intrusions of sensory, affective, and cognitive information into conscious awareness or behavior (e.g., dissociative flashbacks), in addition to the inability to access normally accessible information and control motor processes (negative symptoms) (Spiegel and Cardeña, 1991). Dissociation can be conceived as a general tendency (trait dissociation) and a temporary state (state dissociation), and it can also be found in non-clinical populations, but to a considerably lesser extent than those in clinical populations (Waller et al., 1996; Holmes et al., 2005; Butler et al., 2019; Scalabrini et al., 2020).

Following the International Society for the Study of Trauma and Dissociation (2011a,b), five symptoms of dissociation can be identified: depersonalization, derealization, dissociative amnesia, identity confusion, and identity alteration (Sar, 2014). Depersonalization can be described as the sense of being detached from or not feeling in one's body, and it can represent what is often referred to as an “out-of-body” experience (Guralnik et al., 2000; Simeon et al., 2001; Gatus et al., 2022). Derealization is the sense of feeling detached from the world or the sense of the world not being real. It can be an association with a description of the world as being phony, foggy, far away, or as if seen through a veil (Steinberg and Steinberg, 1995; Gatus et al., 2022). Dissociative amnesia can be described as the inability to recall relevant autobiographical information that is not related to ordinary oblivion. Most of the types of amnesia typical of dissociative disorders are not of the classic fugue variety; rather, the different types of amnesias typically refer to important events that are forgotten, such as abuses, accidents, or a limited period of time, ranging from minutes to years (Steinberg and Steinberg, 1995; Staniloiu and Markowitsch, 2014). Identity confusion can be described as a sense of confusion about who a person is. An example of identity confusion is when a person may experience excitement when being involved in an activity that can be found repulsive at other times. Identity alteration can be described as the sense of feeling significantly different from another part of oneself (International Society for the Study of Trauma and Dissociation, 2011a,b; Sar, 2014).

The life-threatening coronavirus disease 2019 (COVID-19) pandemic has been identified as a potentially traumatic event (Gersons et al., 2020) and may produce a wide range of mental health problems (e.g., depression, anxiety disorders, sleep disorders, and dissociation) stemming from pandemic-related events, such as sickness, isolation, losing loved ones, and fear for one's life (El-Hage et al., 2020; Mækelæ et al., 2020; BinDhim et al., 2021). Moreover, Horesh and Brown (2020) concluded that COVID-19 should be viewed from the perspective of trauma because it will most certainly lead to stress-related mental health issues. In adults, COVID-19-related events have been shown to be associated with more severe trauma-related symptoms than other potentially traumatic events (Olff et al., 2021; Havermans et al., 2023). Structural dissociation of the personality (SDP) theory (Van Der Hart et al., 2004; van der Hart et al., 2006) and polyvagal theory (Porges, 2007, 2022) may promote the conceptualization of COVID-19 pandemic-triggered dissociative disorders within a psychophysiological and neurophysiological framework. In addition, eye movement desensitization and reprocessing (EMDR) therapy has long been shown to be effective with post-traumatic stress disorder (PTSD) (Moreno-Alcázar et al., 2017) and beyond PTSD (Valiente-Gómez et al., 2017), as with trauma-related psychological disorders (Perlini et al., 2020), such as dissociative disorders (Mitra and Jain, 2023). Accordingly, EMDR therapy has been successfully used in treating traumatic stress symptoms of COVID-19 patients (Brennstuhl et al., 2022; Yurtsever et al., 2022).

Therefore, the objective of this study is two-fold: (1) elucidating the contribution of the SDP theory and polyvagal theory to the conceptualization of the COVID-19 pandemic-triggered dissociative disorders and the importance of assessing perceived safety in dissociative disorders through neurophysiologically informed psychometric tools and (2) analyzing the contribution of the EMDR to the treatment of the COVID-19 pandemic-triggered dissociative disorders and suggesting possible neurobiological mechanisms of action of the EMDR.

## 2. Dissociative disorders and structural dissociation of the personality: evidence from psychotherapy and neuroscience

**Figure 1:**
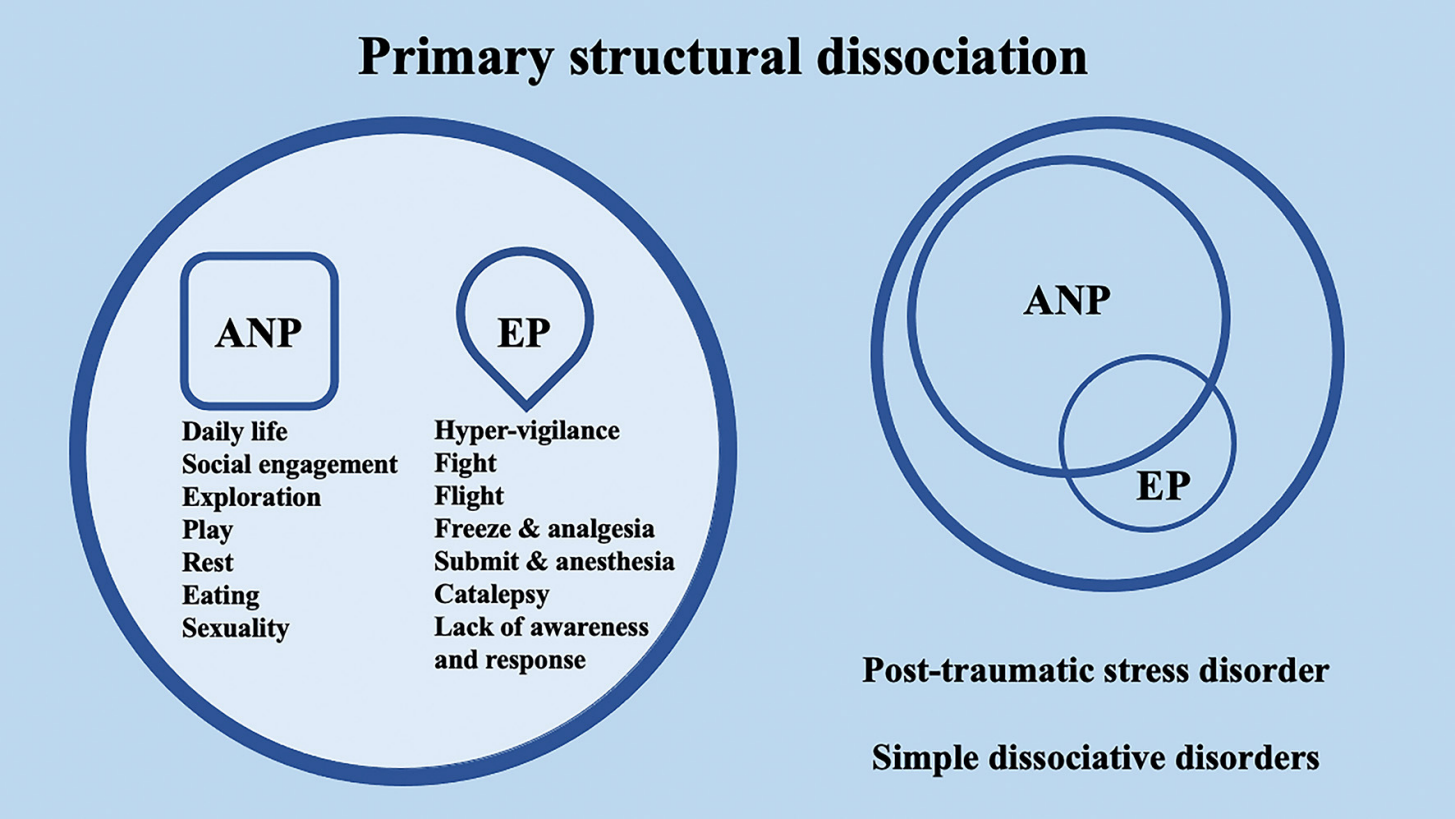
Schematic representation of primary structural dissociation, mainly regarding post-traumatic stress disorder (PTSD) and simple dissociative disorders, according to the structural dissociation of personality (SDP) theory.

**Figure 2:**
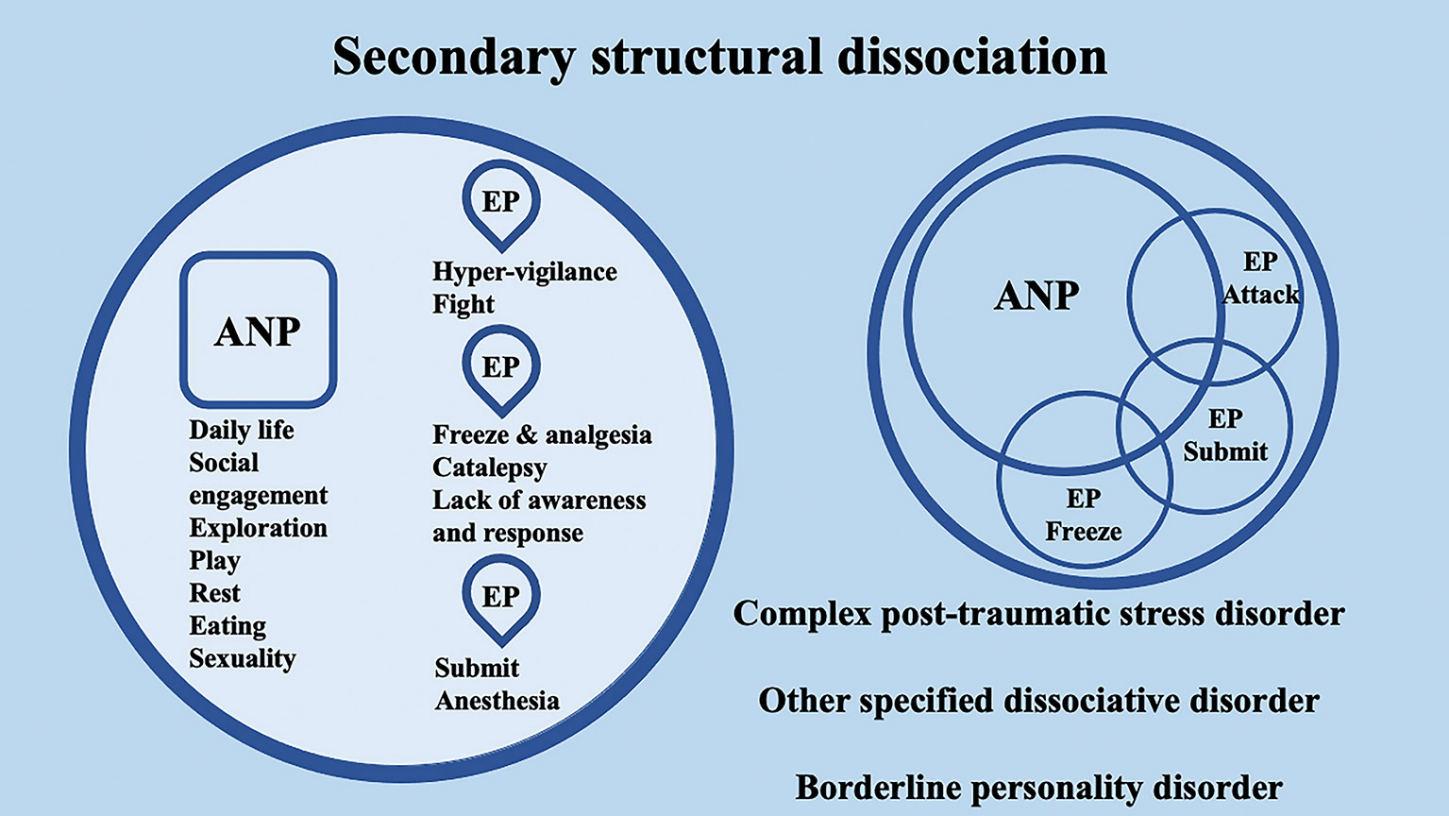
Schematic representation of secondary structural dissociation, mainly regarding complex post-traumatic stress disorder (C-PTSD), BPD, and other specified dissociative disorders (OSDD), according to the structural dissociation of personality (SDP) theory.

**Figure 3:**
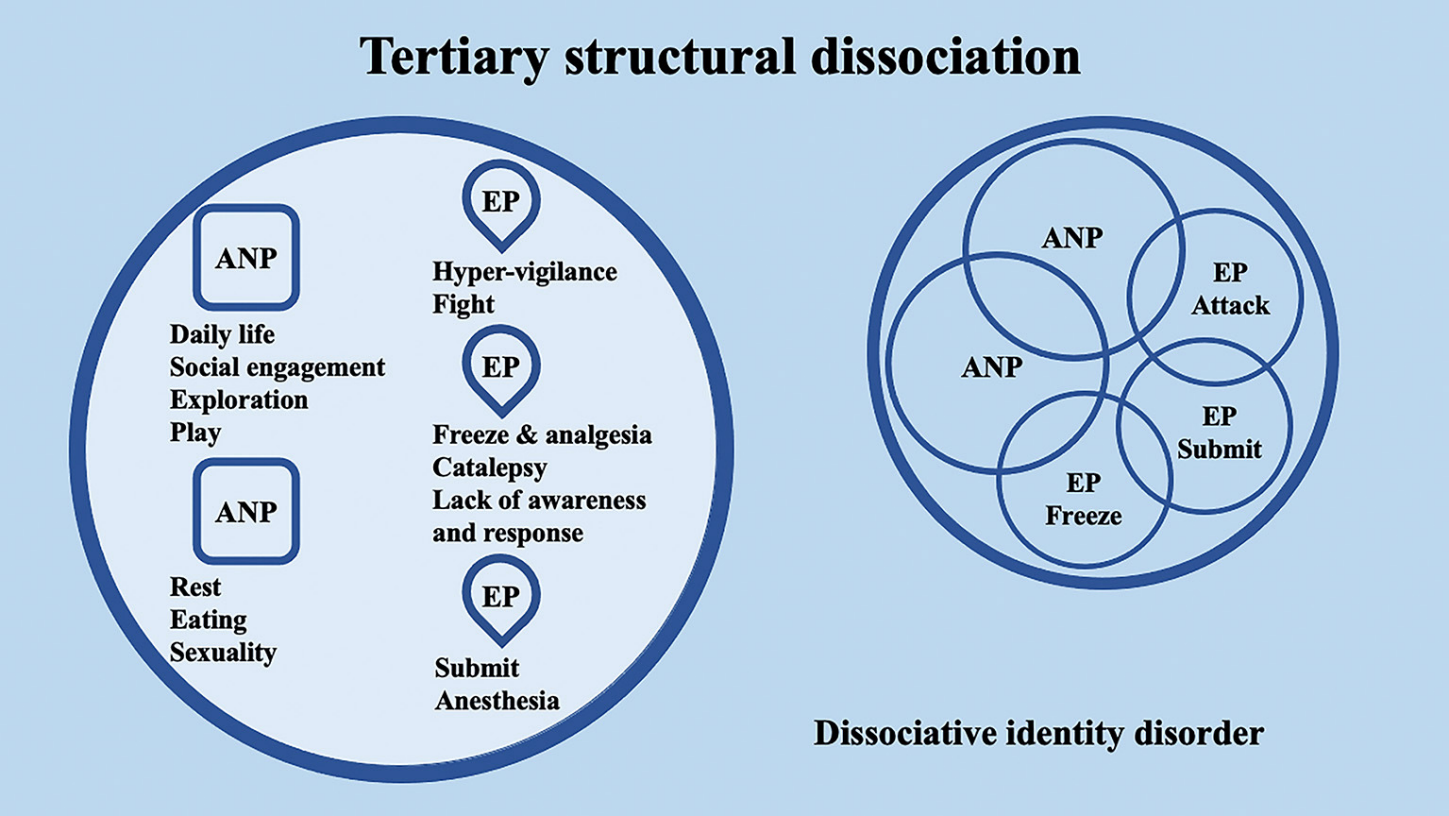
Schematic representation of tertiary structural dissociation, mainly regarding DID, according to the structural dissociation of personality (SDP) theory.

According to the conceptualization of SDP (Van Der Hart et al., 2004; van der Hart et al., 2006), Dissociative identity disorder (DID) patients have “apparently normal part(s) of the personality” (ANPs, responsible for coping with the demands of daily life) and “emotional part(s) of the personality” (EPs, fixated in traumatic memories, which assure survival in situations of severe threat). According to the SDP theory, individuals with primary structural dissociation show one ANP and an EP (Figure 1), and this structure is believed to be reflected in PTSD and simple dissociative disorders (e.g., depersonalization/derealization disorder or dissociative amnesia). In primary structural dissociation, a unique EP is presumed to handle fight, flight, freeze, and submission (Van Der Hart et al., 2004; van der Hart et al., 2006). When an individual shows multiple EPs and one ANP, this condition is referred to as secondary structural dissociation (Figure 2). Analogously to primary structural dissociation, in secondary structural dissociation, the ANP is responsible for daily activities, while the multiple EPs retain traumatic information that the ANP was unable to effectively integrate. However, unlike primary structural dissociation, each of the multiple EPs addresses distinct and frequently conflicting parts of the trauma. Different EPs will be accompanied by various memory clusters, strong emotions, learned behaviors, internalized messages, and personality traits. The multiple EPs may also be more structured and developed than in primary structural dissociation. Complex-PTSD (C-PTSD), Borderline personality disorder (BPD), or other specified dissociative disorder (OSDD) may arise when some or all of these criteria are present (Cloitre et al., 2014). The existence of multiple ANPs and multiple emotionally distinct EPs within an individual is referred to as tertiary structural dissociation (Figure 3). Each of the ANPs involved in tertiary structural dissociation deals with distinct (though potentially overlapping) facets of daily life. One ANP might be the host and manage studies, relationships, and employment. Another ANP might be better at relationships and help the first in that regard, either knowingly or unknowingly. Another ANP might assist with a particular subject matter in school or an aspect of work. Patients with DID lack a single ANP that perfectly captures who they would be as a fully unique developed personality. Before people with DID become aware of their condition, their ANPs are extremely phobic of their EPs, while some ANPs can search for less-frightening EPs once they are aware of what the EPs are. Alters (i.e., alternate identities that can be classified as either ANPs or EPs) can be highly developed and structured and have strong dissociation boundaries between them, so ANPs can frequently interact safely with specific EPs without necessarily re-experiencing traumatic memories, perceptions, or cravings (van der Hart et al., 2006). ANPs may use amnesia, anesthesia, a narrowing of the ANPs' emotional range, or numbing of their emotional intensity as preventative measures to hinder the activation of EPs. These avoidant actions, when coupled with EPs' frequent intrusions, might deplete mental resources and promote depression, anxiety, or persistent feelings of helplessness, shame, guilt, or rage. To firmly anchor themselves to the present and stop EPs from intruding, particularly desperate ANPs may self-harm or utilize psychoactive substances, while emotionally distant ANPs may find it difficult to build long-term relationships. Theoretically, tertiary structural dissociation is the same as DID; however, actually, some patients with OSDD may meet the criteria, while other patients with DID may not because of the insufficient number of parts they show. There is evidence that adults with DID may develop additional EPs or additional ANPs depending on their emerging needs and experiences. While new actual or perceived trauma is most likely to result in new EPs, new ANPs can also emerge to handle new work challenges or to replace previous ANPs who are no longer able to handle daily routines (Van Der Hart et al., 2004; van der Hart et al., 2006).

There is evidence that this hypothesized structural dissociation is also reflected psychophysiologically. Reinders et al. (2006) demonstrated that there are significant differences in psychophysiological reaction [e.g., heart rate (HR), heart rate variability (HRV)] to a trauma script between “neutral identity states” (i.e., ANPs), which were distinguished by a blunted psychophysiological reactivity to the trauma script, and “traumatic identity states” (i.e., EPs), which presented a higher HR and lower HRV (Reinders et al., 2006). Furthermore, to investigate the hypothesis that ANPs and EPs in DID have different perfusion patterns in response to rest instructions and that perfusion is different in actors who were instructed to mimic ANPs and EPs, arterial spin-labeling perfusion MRI was utilized (Schlumpf et al., 2014). ANPs showed increased thalamic perfusion compared to EPs. The dorsomedial prefrontal cortex, primary somatosensory cortex, and motor-related regions were found to show higher perfusion in EPs compared to ANPs. Mimicked ANPs' and EPs' perfusion patterns were different. In line with their reported role-playing techniques, the brains of individuals who were instructed to mimic ANPs and EPs activated regions that were related to visual mental imagery and experienced empathy. Dissociative part-dependent differences in resting states characterize DID. The EPs generated increased activation in brain regions involved in self-referencing and sensorimotor activities compared to ANPs. Actors' perfusion patterns were different from those of real ANPs and EPs. Hence, the authors conclude that the resting-state properties of ANPs and EPs in DID are not caused by suggestion, fantasy proneness, and role-playing (Schlumpf et al., 2014). In addition, fewer omission errors during an n-back working memory task were made by DID simulators with respect to individuals with an actual DID. Regarding the prefrontal parietal network, the simulated neutral states, as well as the trauma-related identity states of DID simulators, but not those who had actual DID or PTSD, activated working memory related to the left frontal pole and ventrolateral prefrontal cortex (Brodmann area 44) (Vissia et al., 2022). Notably, using arterial spin-labeling perfusion MRI in a large cohort of 59 patients with severe COVID-19, hyperperfusion clusters were detected near motor and premotor cortices and in parietotemporal regions (Ardellier et al., 2023). Interestingly, motor and premotor cortices are prefrontal, motor-related regions, and hyperperfusion in these regions may parallel the pattern of activation found by Schlumpf et al. (2014) during EPs. In accordance with this, hyperperfusion in the parietotemporal regions is related to the primary somatosensory cortex and regions related to memory and self-referencing. Hence, the parietotemporal hyperperfusion found in severe COVID-19 patients by Ardellier et al. (2023) may parallel the pattern of activation found by Schlumpf et al. (2014) during EPs as well. Taken together, this evidence suggests that severe COVID-19 may impinge on brain regions that are also involved in EPs' activation, promoting the emergence of dissociation and dissociative symptomatology. In addition, using arterial spin-labeling MRI in 24 patients with persistent cognitive complaints in the post-COVID-19 period (known as long-COVID), Ajčević et al. (2023) reported a significant hypoperfusion predominantly affecting the frontal cortex, as well as the parietal and temporal cortices. The hypoperfusion areas identified in the right hemisphere regions were more extensive. Overall, a hyperperfusion of prefrontal–parietotemporal regions during severe COVID-19, possibly triggering autobiographical memories, EPs, and dissociative symptoms, may lead to a hypoperfusion of the frontal executive network in the post-COVID-19 period, generating cognitive impairment. In accordance with this, it has been proposed that when areas pertaining to the autobiographical memories are active (i.e., the default mode network), regions related to executive function (i.e., central executive network) deactivate (Menon and Uddin, 2010; Menon, 2011). Intriguingly, Parlar et al. (2016) reported that dissociative symptoms may act as a transdiagnostic factor associated with neuropsychological dysfunction in 23 patients with a history of trauma and depression. Taken together, these results suggest that Severe Acute Respiratory Syndrome COronaVirus 2 (SARS-CoV-2), causing severe COVID-19, may retain the potential to disrupt the activity of brain regions related to autobiographical memories, triggering EPs and dissociative symptoms that, in the long term, may disrupt cognitive performance, in the long-COVID period. In fact, it has been reported that SARS-CoV-2 shows a neuroinvasive potential (Li et al., 2020) that may contribute to respiratory failure of COVID-19 patients and may promote the disruption of the activity of brain regions related to autobiographical memories that will be further discussed in light of the polyvagal theory (Poli et al., 2020).

## 3. Integrating the polyvagal perspective in the conceptualization of dissociative disorders through structural dissociation of the personality

According to the polyvagal theory, the evolutionary roots of the human autonomic nervous system have generated a hierarchical structural and functional organization (Porges, 2007, 2009, 2022). The social engagement system (SES) is derived from the myelinated ventral vagal complex (VVC), whose cardioinhibitory fibers emerge from the nucleus ambiguus (NA) in the brainstem (SES). The VVC is the least disruptive to homeostasis, phylogenetically youngest, and fastest responding challenge–response system due to its myelinated fibers. Phylogenetically older than the VVC, the sympathetic nervous system (SNS) supports increased breathing, HR, and mobilized behaviors in order to implement aggressive reactions to dangers, such as flight or defensive fight. The dorsal motor nucleus of the vagus (DMNX) in the brainstem is the region where the unmyelinated cardioinhibitory fibers that generate the dorsal vagal complex (DVC), the phylogenetically oldest autonomic branch, originate. Additionally, it has a vestige immobilization ability that first appeared in early vertebrates (Porges, 2021a). Organs below the diaphragm are innervated by the DVC, which also plays a role in homeostatic and threat responses. This complex, when activated during stress responses, also impairs digestion and preserves metabolic resources (Porges, 2007, 2009). Notably, the DVC is the system that plays a major role in post-traumatic reactions following psychological trauma (Kolacz and Porges, 2018; Kolacz et al., 2019).

According to the polyvagal theory, the integration of myelinated cardiac vagal pathways with neuronal control of the face and head promoted the emergence of the mammalian SES. Early abnormal coordination of this system is a predictor of future emotional regulation and social conduct problems. The preferential recruitability of the SES, or the sequential hierarchical recruitment of the SNS, or the DVC is determined by the brain's evaluation of environmental risk. According to the polyvagal theory, neuroception—a neural reflexive mechanism that is distinct from perception and capable of instantly altering the physiological state and differentiating between safe, dangerous, and life-threatening environmental and visceral features—is used to achieve the neural evaluation of risk. The autonomic state is adaptively modified to lower SNS activity and protect the oxygen-dependent central nervous system, particularly the cortex, from the metabolically conservative reactions of the DVC (e.g., fainting), and a neuroception of safety supports the SES in safe environmental conditions. In contrast, the recruitment of SNS or DVC is favored by a neuroception of danger or a life threat (Porges, 2003, 2007, 2009, 2021b).

**Figure 4:**
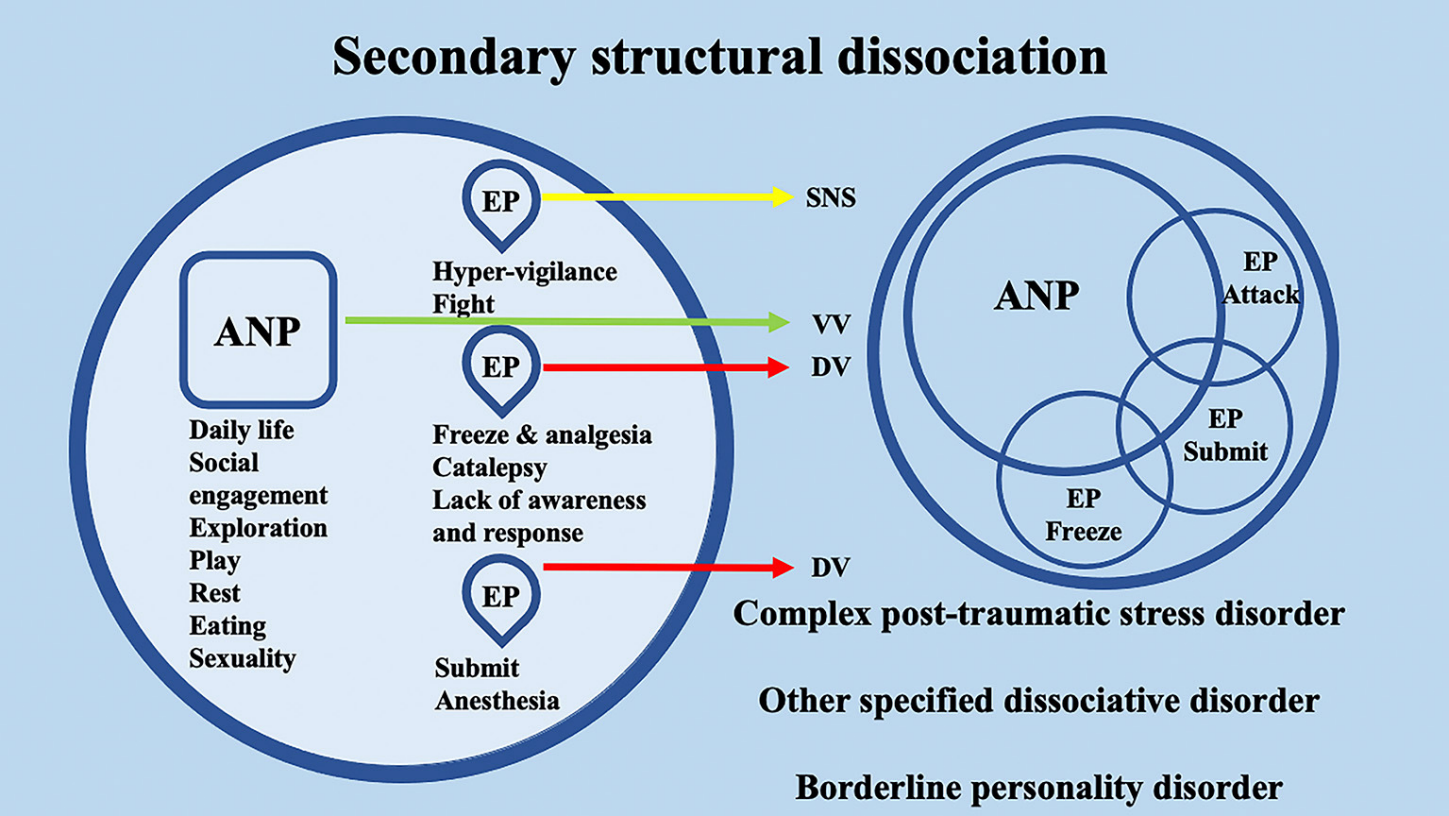
Schematic representation of the polyvagal conceptualization of secondary structural dissociation according to the structural dissociation of personality (SDP) theory.

**Figure 5:**
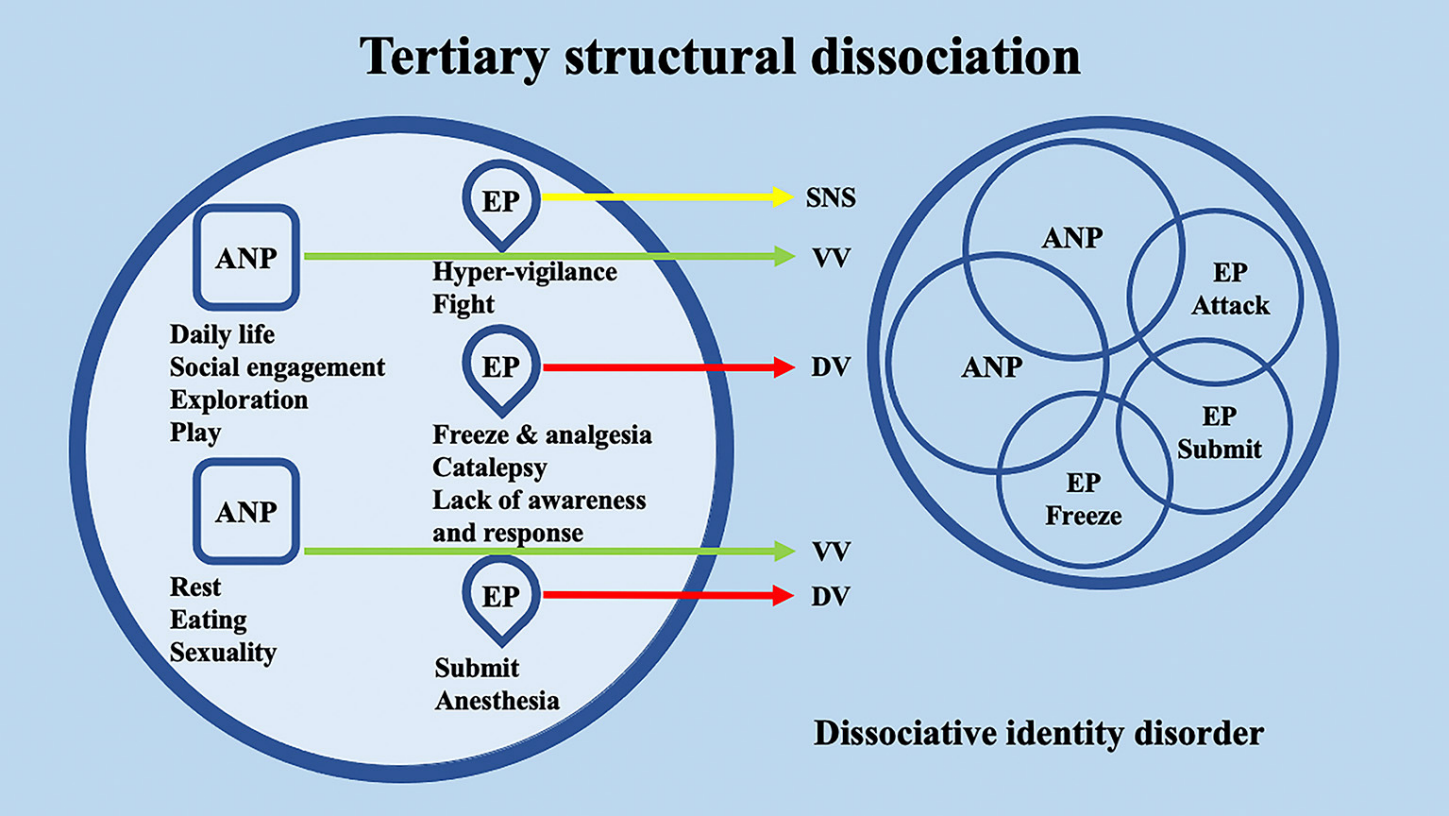
Schematic representation of the polyvagal conceptualization of tertiary structural dissociation according to the structural dissociation of personality (SDP) theory.

Interestingly, the conceptualization of DID through the SDP theory confers to the ANPs' and EPs' specific behaviors and neurophysiological responses. Through the lens of the polyvagal theory, these responses may be differentially supported by the SNS or by the two different branches of the Parasympathetic nervous system (PNS) (i.e., VVC or DVC). For example, in secondary structural dissociation, mainly regarding C-PTSD, BPD, and OSDD, a unique ANP is responsible for daily activities, while the multiple EPs retain traumatic information that the ANP was unable to effectively integrate. In particular, ANP is supposed to deal with daily routine activities, social engagement, exploration, play, rest, eating, and sexuality and may be mainly supported at the neurophysiological level by the VVC. Among multiple EPs, an EP may specifically implement hypervigilant neurophysiological reactions and fight behavioral responses; hence, it may be mainly supported at the neurophysiological level by the SNS. A second EP may implement freezing and analgesia responses, catalepsy, and lack of awareness and response, while a third EP may implement responses related to submission and anesthesia. The neurophysiological and behavioral responses of the second and third EPs may be mainly supported at the neurophysiological level by the DVC (Figure 4). Analogously, in tertiary structural dissociation, mainly regarding DID, multiple ANPs and multiple emotionally distinct EPs are supposed to exist. The main difference with respect to secondary structural dissociation is the possible presence of multiple ANPs. A first ANP may deal with daily routine activities, social engagement, exploration, and play, while a second ANP may be responsible for implementing rest, eating, and sexuality. Both of these ANPs may be mainly supported at the neurophysiological level by the VVC, though there may be no integration between them (Figure 5).

Interestingly, SARS-CoV-2 viral antigens have been detected in the brainstem, where the infected regions included the nucleus of the solitary tract (the main input source for both DMNX and NA in the polyvagal theory) and NA (Li et al., 2020), whose myelinated fibers contribute to VVC, health, and social engagement, potentially characterizing COVID-19 as a cardiorespiratory disease (Poli et al., 2020). Disrupting the activity of the nucleus of the solitary tract and NA in the brainstem through its neuroinvasive potential (Lahiri et al., 2020; Li et al., 2020; Yashavantha Rao and Jayabaskaran, 2020; Valizadeh et al., 2022), SARS-CoV-2 may foster the perturbation of the SES and promote the activation of the cortical regions supporting the emergence of autobiographical EPs, possibly supported by SNS or DVC, and dissociative symptoms.

## 4. EMDR as a psychotherapeutic integrative strategy promoting psychological and neurophysiological integration in dissociative disorders

The World Health Organization (WHO) has identified the EMDR, an evidence-based psychotherapy [World Health Organization (WHO), 2013], as a first-choice treatment for PTSD. The EMDR was rated as strongly recommended by the new International Society for Traumatic Stress Studies guidelines (Bisson et al., 2019) for the treatment of PTSD in children, adolescents, and adults. The National Institute for Health and Care Excellence guidelines, the aforementioned WHO recommendations, high-quality systematic reviews carried out through the Cochrane database, and the outcomes of randomized-controlled trials served as the foundations for these recommendations. The effectiveness of EMDR in treating different mental and somatic diseases that are also accompanied by psychological trauma has been the subject of growing research over the past 10 years (Moreno-Alcázar et al., 2017; Valiente-Gómez et al., 2017; Perlini et al., 2020; Gielkens et al., 2022; Onofri, 2023). The adaptive information processing (AIP) paradigm, on which the EMDR is based, states that overwhelming and traumatic adverse childhood experiences are often maladaptively encoded and/or incompletely processed, and, as a result, contribute significantly to psychopathology (Hase et al., 2017; Poli et al., 2022a,c). Two recent basic studies have significantly explored and elucidated the neurobiological mechanisms underlying the EMDR. One seminal study, carried out in mice (Baek et al., 2019; Maddox et al., 2019), describes the neuroanatomical pathway and mechanism of action of the EMDR. By pairing visual alternate bilateral stimulation (ABS) with conditioned stimuli during fear extinction, the authors were able to successfully induce a long-lasting decrease in fear in mice. ABS showed the greatest impact on lowering fear and promoted a long-lasting increase in the superior colliculus (SC) and the mediodorsal thalamic (MDT) activity. In fact, basolateral amygdala inhibitory neurotransmission was stabilized, and fear-encoding cell activity was reduced, by ABS via a feedforward inhibitory circuit stemming from the MDT. Most importantly, using optogenetic manipulation, it was found that the SC-MDT circuit was both necessary and sufficient to prevent the return of fear (Baek et al., 2019). The second study highlights the EMDR as a successful psychotherapy for rewriting traumatic memory engrams, which serve as the basis for traumatic memory persistence after the encoding of the threat experience in the brain circuits (Maddox et al., 2019).

Interestingly, it has been shown that the EMDR intervention was able to exert a therapeutic effect through the promotion of homeostasis in the autonomic nervous system. In 10 patients with PTSD, it was shown that, at ABS onset, a significant decrease in HR and a rapid increase in HRV were observed, both of which indicated an arousal decrease and a significant PNS effect. During ongoing ABS, pre-ejection period and HRV significantly diminished, while the respiration rate significantly increased, indicating stress-related arousal and SNS significant effects. However, over the course of sessions, a remarkable reduction in psycho-physiological activity was observed, as shown by a gradual decrease in HR and an increase in HRV, indicating a generally significant PNS effect. These results suggest that the EMDR may promote a reprocessing of traumatic memories recruiting PNS activity that may be able to promote a homeostatically balanced activity of SNS and of the autonomic nervous system as well (Sack et al., 2008).

As discussed previously, ANPs and EPs in DID may have different psychophysiological responses (Reinders et al., 2006; Schlumpf et al., 2014, 2021; Vissia et al., 2022). Furthermore, it has been argued that HRV, electroencephalography, and fMRI are sensitive techniques to identify physiological changes linked to dissociation and dissociative disorders, and they may be able to elucidate their possible origin. These investigations may eventually lead to the development of biomarkers that may be used to precociously identify at-risk patients and treat dissociative disorders appropriately (van der Kruijs et al., 2014; Vesuna et al., 2020; Feinstein and Voon, 2021; Modesti et al., 2022; Arancibia et al., 2023). Interestingly, according to the polyvagal theory, respiratory sinus arrhythmia (RSA) reflects the activation of the VVC (Porges, 2009; Poli et al., 2021a, 2022c) and has been proposed as a transdiagnostic biomarker of stress vulnerability across psychopathologies (Beauchaine, 2015; Beauchaine and Bell, 2020). Diminished RSA and hypertrophic RSA reactivity (i.e., withdrawal) to emotional challenge are consistently observed among individuals with impaired emotion regulation (ER) capacities, namely, those with various forms of internalizing and externalizing psychopathology, such as anxiety, phobias, attention problems, autism, dysphoria, conduct disorder, depression, non-suicidal self-harm, panic disorder, and trait hostility. There is emerging evidence that diminished RSA and hypertrophic RSA reactivity may index poor ER because they are downstream markers of prefrontal cortex dysfunction (Beauchaine and Bell, 2020).

Notably, EMDR treatment has been associated with increased RSA (Sack et al., 2008), and, therefore, according to the polyvagal theory, the EMDR may promote VVC. In fact, according to the evidence, successful traumatic memory resolution may be linked to an increase in parasympathetic tone, both when PTSD patients were confronted with a reminder of the experience and while in a baseline control state. The observed RSA increase under both control and trauma script conditions suggests stronger parasympathetic tone levels, and in particular VVC levels, following EMDR therapy, which may be related to improved abilities to control psychophysiological stress reactions and to improved ER. According to the AIP paradigm (Shapiro and Laliotis, 2011; Shapiro, 2014, 2018), successful EMDR therapy of implicit traumatic memory may promote the integration of the somatic component of the experience, resulting in a decrease in physiological reactivity. Research evidence suggested that EMDR therapy may be able to promote psychophysiological regulation—during exposure to a traumatic memory trigger—as well as a decline in psychobiological markers of chronic stress (Sack et al., 2008; Campbell et al., 2019). Furthermore, these results are consistent with the theory that trauma integration by EMDR therapy may result in the restoration of myelinated VVC cardioinhibitory fibers that may be able to downregulate the DVC-generated traumatic response restoring an internal neuroception of safety (Poli et al., 2022a,b,c; Porges, 2022). In accordance with this, it has been shown that EMDR-related visual ABS shifted the autonomic balance as indicated by decreases in HR, skin conductance, and the ratio between the low- and high-frequency components of the HR power spectrum (LF/HF) and an increased finger temperature. Overall, these findings suggested that eye movements during the EMDR activate cholinergic PNS and inhibit the SNS (Elofsson et al., 2008). In addition, this evidence is not in contrast with the phase-oriented treatment of structural dissociation (Steele et al., 2005; Horowitz, 2022). Phase 1, symptom reduction and stabilization, is geared toward overcoming phobias of mental contents, dissociative parts, and attachment and attachment loss with the therapist. Phase 2, treatment of traumatic memories, is directed toward overcoming the phobia of traumatic memories and phobias related to insecure attachment to the perpetrator(s), particularly in EPs. In phase 3, integration and rehabilitation, treatment is focused on overcoming phobias of normal life, healthy risk-taking and change, and intimacy. In particular, the EMDR can be integrated into phases 2 and 3 but also into phase 1 in terms of desensitization and resource installation (Shapiro, 2018; Stingl et al., 2022).

## 5. Possible neural mechanisms of action of the EMDR and polyvagal theory

**Figure 6:**
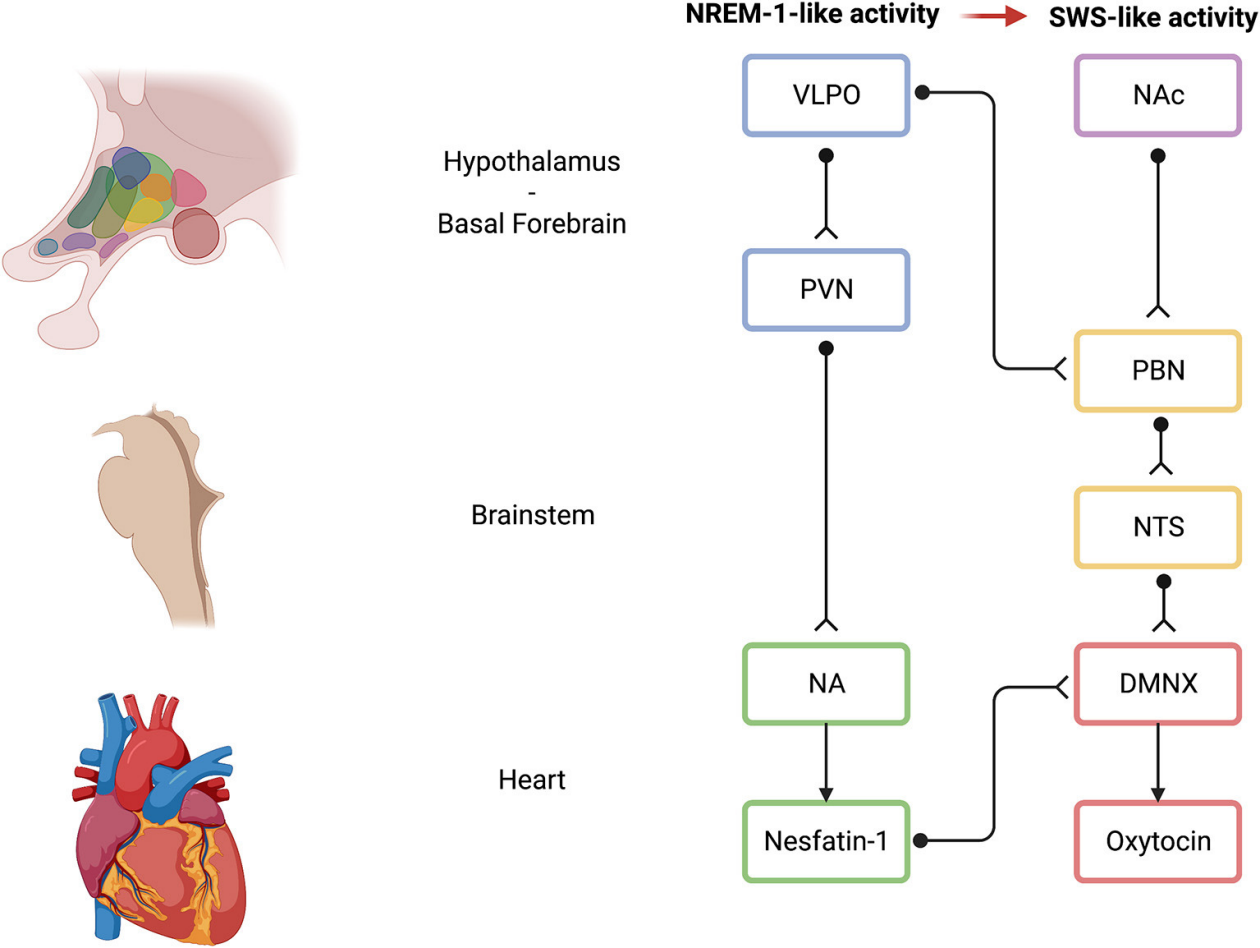
Possible neural mechanisms of action of the EMDR induced by ABS. Light blue indicates hypothalamic brain areas; pink indicates basal forebrain brain areas; light yellow indicates brainstem brain areas; light green indicates brainstem-related NA activity on the heart and on the DMNX; and light red indicates brainstem-related DMNX activity. Black unipolar neurons indicate a schematic representation of neuronal connections among brain areas. VLPO, ventrolateral preoptic area; NAc, nucleus accumbens; PVN, paraventricular nucleus; PBN, parabrachial nucleus; NTS, nucleus of the solitary tract; NA, nucleus ambiguus; DMNX, dorsal motor nucleus of the vagus; EMDR, eye movement desensitization and reprocessing; ABS, alternate bilateral stimulation. Created with BioRender.com.

In addition to the aforementioned evidence provided by Baek et al. (2019), some evidence exists that links the EMDR-related visual ABS to the parasympathetic-related VVC and polyvagal theory. Typical visual ABS of the EMDR ranges between ~1 and 2 Hz (Pagani et al., 2017) and are similar to slow eye movements (SEMs) that manifest at human sleep onset and that are conjugate, regular, sinusoidal, primarily horizontal eye movements that range in amplitude from moderate to large (Pizza et al., 2011), and sleep disturbances are considered a hallmark of PTSD (Germain, 2013). SEMs are known to occur at sleep onset, during the transition between wakefulness and sleep, as well as during non-rapid-eye-movement (NREM) sleep stage 1 (NREM-1) (Porte, 2004). Interestingly, during NREM sleep, sleep-active neurons in the ventrolateral preoptic area (VLPO) are thought to synapse with the hypothalamic paraventricular nucleus (PVN) that, in turn, project to NA/DMNX (Silvani et al., 2018). In addition, in mice, slow-wave sleep (SWS) has been shown to be controlled by a subset of nucleus accumbens (NAc) core neurons as well (Oishi et al., 2017), and NAc has long been known to project to the parabrachial nucleus that, in turn, projects to the nucleus of the tractus solitarius (NTS), an important brain region according to the polyvagal theory (Lazarus et al., 2012). NTS successively projects to NA/DMNX as well. Notably, in cats, NA and DMNX were stimulated with and without cardiac pacing, before and after ipsilateral vagotomy. DMNX stimulation produced no changes in HR, while HR decreased after NA stimulation. Overall, DMNX may control ventricular contractility, whereas NA may be involved in HR control (Geis and Wurster, 1980). Interestingly, it has been shown that optogenetic activation of channelrhodopsin-2-expressing PVN neurons in the brainstem activated oxytocin receptors in the DMNX. Most importantly, activation of oxytocin receptors was found to mediate both sustained enhancement of glutamate release and short-term synaptic plasticity-related paired pulse facilitation (Piñol et al., 2014). Analogously, it has been shown that nesfatin-1 was able to activate NA cardioinhibitory fibers, confirming the effect of a decrease in HR not accompanied by a change in blood pressure (Geis and Wurster, 1980; Brailoiu et al., 2013). Nesfatin-1 was discovered in 2006 in the rat hypothalamus as an 82-amino acid polypeptide, cleaved from the 396-amino acid precursor protein nucleobindin-2, and was first highlighted for its anorexigenic effects (Oh-I et al., 2006). Subsequently, other functions of nesfatin-1, such as cardiovascular effects, lipid metabolism, reproduction functions, and emotion-related functions, were found (Gao et al., 2016; Prinz and Stengel, 2016; Wei et al., 2018). However, the receptor mediating these effects and the exact signaling cascades remain still unknown even if recent data suggest that a possible signaling pathway may be represented by a G protein-coupled receptor (Rupp et al., 2021). Interestingly, upstream of the information processing, nesfatin-1, at the mRNA and protein level, was found in the preoptic area, NAc, and NA, as well (Goebel et al., 2009). Most importantly, it has been shown that PVN-generated, NA-related, nesfatin-1 was able to support DMNX-related oxytocin signaling function and, in particular, that nesfatin-1 could be an upstream regulator of oxytocin (Nakata et al., 2016). Nesfatin-1 could possibly be an upstream regulator of DMNX-related oxytocin due to its myelinated fibers (Li et al., 2020; Poli et al., 2021a, 2022c). In addition, NAc may represent a critical brain region for the therapeutic effects induced by the EMDR through SEM: it has been demonstrated that NAc can orchestrate the SWS (Oishi et al., 2017), but it has long been known that NAc is a critical brain region that is included in the mesolimbic dopamine pathway regulating social attachment (Opendak et al., 2019). In particular, mesolimbic dopamine pathway (e.g., midbrain ventral tegmental area projecting to NAc) is implied in the maternal regulation of infant fear that is disrupted by maltreatment and adverse childhood experiences (Opendak et al., 2019). The DVC, which retains traumatic memories (Kolacz and Porges, 2018; Kolacz et al., 2019), contains the NTS, the DMNX, and area postrema. It has been shown that the NTS was able to project to the PVN (that is downstream to NREM-1-like EMDR SEMs-activated VLPO area) and to the NAc (Keller et al., 2022). As mentioned before, NAc is also able to project to the parabrachial nucleus that, in turn, projects to the NTS (Lazarus et al., 2012). Overall, VLPO neurons may be activated by the EMDR-induced SEMs, generating the emergence of NREM sleep stage 1-like neuronal activity, and, downstream through the PVN, NA-related nesfatin-1 may regulate the activation of DMNX-related oxytocin (Figure 6). However, this initial NREM-1-like activity may facilitate the re-emergence of DVC-related traumatic memories, feelings, and early attachment injuries, where NAc may be increasingly involved, and that may typically pertain to SWS, NREM stages 3 and 4. Hence, in the presence of a neuroception of safety within the clinical setting (Porges, 2003), the patient may be able to tolerate uncomfortable feelings and maintain these feelings within a window of tolerance. The presence of safety may promote the maintenance of oxytocinergic neurotransmission (Uvnäs Moberg et al., 2022) that, in turn, may foster the functional coupling between PVN and both sympathetic and parasympathetic cardioinhibitory nuclei (Yee et al., 2016). In fact, previous research showed that oxytocin treatment resulted in functional coupling between PVN activity and brain regions regulating both sympathetic (i.e., rostral ventrolateral medulla) and parasympathetic (i.e., DMNX and NA) branches of the autonomic nervous system (Yee et al., 2016). Therefore, during the EMDR therapy sessions, the presence of safety may promote the emergence of adaptive sympathetic neurophysiological responses (e.g., anger and compelling feelings to attack or to escape) that may contribute to the traumatic memory desensitization and reprocessing rather than the emergence of dysfunctional sympathetic responses when a lack of safety is present. During traumatic memory processing, the visual ABS progression may promote the achievement of the most evolved neurophysiological step related to VVC and SES, which are supported by NA.

## 6. Neurophysiologically informed psychometric tools to assess perceived safety in dissociative disorders

Since traumatic symptoms emerge from uncontrolled threat concerns when safety and self-regulation are lacking (Motsan et al., 2021), it is recommended to assess safety in dissociative disorders using psychometric tools with reliable psychometric properties that are rooted on how the autonomic nervous system is structured. Past research has restricted the use of psychometric measures of feeling safe to specialized contexts, such as team safety (Edmondson, 1999), a childhood memory of safety (Richter et al., 2009), as a subscale (Veale et al., 2016), or as a dimension of a broader scale under factor analysis (Gilbert et al., 2008), rather than the central construct. From a methodological perspective, it is recommended to simultaneously measure the effects of many physiological systems on psychological outcomes, as well as to take into account the role of developmental history in better understanding the link between physiology and behavior (Hagan et al., 2020). Considering the importance of safety evaluation within a therapeutic context, recent research has developed new tools to assess feelings of safety. Poli et al. (2021b) identified a 22-item very short form of the 46-item body perception questionnaire-short form (Cabrera et al., 2018), the BPQ-22, consisting of three factors, namely, body awareness (BOA), supradiaphragmatic (SUP), and BOA/subdiaphragmatic (BOA/SUB). The BOA factor is related to items pertaining to the awareness of functions of the upper parts of the body (e.g., “watering or tearing of my eyes”) or of the whole body (e.g., “goosebumps”), while the SUP factor is related to items pertaining to the functions of the upper parts of the body (e.g., “I have difficulty coordinating breathing and eating” and “I have a persistent cough that interferes with my talking and eating”). Interestingly, it was found that the third factor, BOA/SUB, included three items from the original BOA subscale as well as four items that are related to the SUB subscale, all tapping into bloating and digestive issues (e.g., “Stomach distension or bloatedness” and “After eating I have digestive problems”). It has been proposed that a neurophysiological underpinning of the BOA/SUB factor may be represented by the glutamatergic enteroendocrine cells (termed neuropod cells; Kaelberer et al., 2018, 2020) that synapse with pseudounipolar vagal nodose neurons to monosinaptically relay sensory information from the gut to the brainstem, particularly to the nucleus of the tractus solitarius (NTS) (Han et al., 2018; Poli et al., 2021b). NTS is a brainstem nucleus that represents an integrative hub for olfactory and gustatory information (Escanilla et al., 2015) that is upstream to both the NA and the DMNX. In addition to receiving sensory information also from the gut, the NTS also receives information from the insula (Kawai, 2018) that is typically believed to play a critical role in human interoceptive awareness (Craig, 2009; Paulus and Khalsa, 2021). Thus, the NTS seems to integrate subdiaphragmatic reactivity information originating directly from the gut (e.g., the information outflow originating from neuropod cells) and bodily awareness information originating from the insula. Accordingly, the SUB component of our BOA/SUB factor may be represented by gut sensory information projected by cells, such as neuropod cells, and relayed by NTS to cardioinhibitory fibers stemming from the DMNX, while the BOA component may be represented by bodily awareness information projected by insula and relayed by NTS to cardioinhibitory fibers stemming from NA. Together, this may represent the implementation of the immobilization without fear state, through a neuroception of safety (Porges, 2021b), that, in the polyvagal theory, is believed to require a co-activation of the NA and the DMNX fibers. The co-activation of myelinated NA fibers would assure a sense of safety given by the awareness of one's own bodily state, which could be, or promote, a portal to self-compassion (Porges, 2017; Di Bello et al., 2020).

Morton et al. (2022) developed the neuroception of psychological safety scale (Italian version in Poli and Miccoli, Under Review) that identifies three factors, namely, social engagement, compassion, and body sensations. The first factor (social engagement) is characterized by being accepted, understood, cared for, being able to express oneself without being judged, and having someone to trust. Overall, these items indicated an evaluation of the social environment as non-threatening and safe to engage socially (Poli et al., 2021b; Porges, 2021a,b). The second factor (compassion) captured items related to the ability to be compassionate and feeling connected, empathetic, caring, and wanting to help. Being compassionate regulated our autonomic nervous system (Kirby, 2017; Kirby et al., 2017), while self-compassion promoted self-regulation through the reduction in symptoms of anxiety, depression, and maladaptive perfectionistic tendencies (Woodfin et al., 2021). Regarding the therapeutic process, compassion is increasingly observed as central to promote safety and self-soothing strategies (Gilbert, 2020). The third factor (body sensations) is related to the internal sensations of the body in the state of calm, capturing the feeling of relaxation in the face and the body, a steady heartbeat and breath, and a settled stomach. The activation and functioning of the SES are associated with the regulatory function, especially of the heart and bronchi and the associated state of relaxation and restoration (Poli et al., 2021b; Porges, 2021a,b).

Interventions that promote safety should be a main area of investigation for future research. Skin-to-skin contact between a mother and her newborn may be a crucial first step in fostering the structure of the baby's physiological systems, namely, stress reactivity, autonomic functioning, and sleep patterns (Feldman et al., 2002; Poli et al., 2022c). From 6 months to 10 years old, skin-to-skin contact improved children's executive and cognitive development as well as autonomic functioning (as determined by the RSA). By the age of 10, kids who had skin-to-skin contact showed a decreased stress response, an enhanced RSA, a more regulated sleep pattern, and a better cognitive function (Feldman et al., 2014). Accordingly, it has been demonstrated in mice that chronic maternal separation (MS) during the first 2 weeks of postnatal life causes a depletion of the oligodendrocyte progenitor pool in MS adults, which is linked to pro-depressive effects and short-term memory impairment. These findings are relevant to the medial prefrontal cortex (mPFC) development. However, chemogenetic neuronal activation normalized the oligodendrocyte progenitor pool in MS animals and prevented the pro-depressive effects and short-term memory impairment, suggesting neuronal activity as an essential treatment for early-life stress and fostering mPFC-related behaviors (Teissier et al., 2020).

## 7. Efficacy of EMDR interventions on the long-term psychological impact of the COVID-19 pandemic

Sleep-related neurophysiological activities may represent the underpinnings promoting the desensitization and reprocessing induced by the EMDR. A 57% prevalence of sleep disturbances as nightmares was found among patients with dissociative disorders (Agargun et al., 2003), and dissociative patients with nightmares experienced a lower amount of SWS (Blaskovich et al., 2020). Recent emerging research posits that COVID-19 can be conceptualized as a traumatic stressor event that can trigger PTSD-like responses [e.g., reactivated World War II traumatic memories during the COVID-19 lockdown period (Bigarré et al., 2021) and exacerbated other related mental health problems (e.g., dissociation)] and supports the “pathogenic event memory” model of traumatic stress (Bridgland et al., 2021; Rutherford et al., 2021). Though there is an emerging need to further examine the effects of online EMDR for PTSD (Lenferink et al., 2020), a study investigating 38 patients, satisfying the Diagnostic and Statistical Manual of Mental Disorders, Fifth Edition (DSM-V) criteria for acute stress disorder, found that a seven-session EMDR therapy was able to reduce anxiety by 30%, traumatic and depressive symptoms by 55%, and maintenance of the effects as indicated by the follow-up evaluation (Perri et al., 2021).

Recent studies have shown that the EMDR may play an important role in public health in relation to the COVID-19 pandemic since recent studies have confirmed its efficacy in COVID-19 patients. Investigating 21 participants hospitalized for COVID-19 after a four-session treatment, the EMDR therapy was shown to be effective in reducing anxiety, depression, intensity of distress, and levels of experienced fear of the unknown related to the COVID-19 pandemic (Brennstuhl et al., 2022). Notably, 38 patients at the earliest stage of distress to manage the ongoing trauma associated with quarantine or COVID-19 disease received a seven-session EMDR therapy that was able to reduce anxiety by 30% and traumatic and depressive symptoms by 55% (Perri et al., 2021). Regarding healthcare workers, a 12-session EMDR therapy proved to be effective at reducing symptoms of depression, burnout, and PTSD, with respect to the control group (Caille et al., 2023). In a sample of 43 healthcare workers from the Nephrology and Dialysis Service, who spontaneously decided to take part in a brief, three-session EMDR treatment, PTSD symptoms showed a significant clinical improvement (Belvedere et al., 2023).

The online EMDR protocol for recent traumas was applied to a sample of 154 individuals working with coronavirus patients, frontline professionals (doctors, nurses, paramedics, and police), relatives of coronavirus patients, coronavirus patients, and relatives of patients who died from coronavirus. PTSD symptom levels were assessed before, after, and 1 month after therapy, and results showed that the online EMDR therapy was effective in reducing the PTSD level in all groups (Yurtsever et al., 2022). A very recent study investigated the efficacy of EMDR therapy as a videoconferencing psychotherapy in 24 frontline mental health workers, exploring the treatment effect on the core characteristics of the trauma memory, namely, subjective disturbance, belief systems, memory intensity, vividness, and levels of emotionality. These results suggested the potential clinical benefits of using the EMDR as a videoconference psychotherapy (Farrell et al., 2022).

A meta-analysis encompassing a total of 11,324 patients showed that the post-COVID-19 syndrome appears to be characterized mostly by fatigue, cognitive impairment (brain fog, memory difficulties, and attention deficit), and sleep abnormalities. Sleep difficulties, anxiety, and sadness are frequent psychiatric manifestations that become progressively more prevalent over time (Premraj et al., 2022). Dissociative disorders are far more common than the majority of professionals realize. In fact, they are more frequent than many psychiatric conditions that are frequently observed in clinical settings. The prevalence of dissociative disorders is up to 46% among clinical samples of inpatients and outpatients (Loewenstein, 2018). Hence, patients with dissociative disorders are frequently misunderstood, mistreated, and undertreated from 5 to 12.4 years. Severe consequences for both individuals and public health result from inadequate diagnosis and treatment, namely, increased functional impairment across domains, disability, health problems, risk of revictimization, suicidal ideation, self-harming behaviors, frequent hospitalizations, and use of health and social services (Boyer et al., 2022). Within this framework, effective intervention strategies to process COVID-19-related traumatic memories, or oldest reactivated traumatic memories, in dissociative patients, and other patients with dissociative symptoms, may retain an elevated priority to dampen individual emotional suffering and reduce public health burden. The EMDR therapy may represent a rapid and effective strategy that can homeostatically regulate the autonomic nervous system of traumatized patients, and, according to the polyvagal theory, the EMDR may be able to promote VVC, emotional regulation, and social connectedness.

## 8. Discussion

Combining the EMDR, SDP theory, and polyvagal conceptualizations with neurophysiological underpinnings, we have highlighted how promoting integration in dissociative disorders through the EMDR may benefit from monitoring the accompanying neurophysiological processes that may be ongoing, along with psychological processing. First, typical visual ABS of the EMDR should not be administered to the patients at a pace exceeding 2 Hz. Stimulation frequency can be easily controlled using light bars for visual EMDR stimulation or through tools for tactile EMDR. Light bars for visual stimulation, or tools for tactile stimulation, can be useful during the psychotherapeutic session in order to respect the patient's neuroception of safety. In fact, during the processing of a very distressful traumatic memory, intrusive and invasive feelings may re-emerge (Ehlers et al., 2002) and patients should be asked whether they prefer the therapist to maintain the same vis-à-vis setting or, for example, to move to a corner of the room. The use of light bars or tools for tactile stimulation maintains the possibility of administering ABS and allows, at the same time, to respect the patient's neuroception of safety. Severely dissociative patients may show aversion to tactile or visual stimulation since vision and touch may have been a sensory system through which psychological or physical abuse was conveyed (Strauss et al., 2019). Hence, though visual ABS has been shown to be more effective in diminishing the emotionality of traumatic memories (van den Hout et al., 2012; de Jongh et al., 2013), with severely dissociative patients, it may be necessary to use auditory ABS.

Second, recently, using multisession functional magnetic resonance imaging, it has been shown that effective updating and reintegration of dysfunctional memories emerged only through a positive emotion-focused strategy (Speer et al., 2021). These results are of particular importance since typical EMDR protocols (Shapiro, 2018) require positive cognition identification. However, different kinds of cognitions may be classified as positive by the patient depending on the nature of the memory to reprocess. For example, if a memory was DVC related, it may be highly probable that the identified positive cognition may be of an SNS nature. In the first instance, this kind of positive cognition may be functional as a resource to overcome the negative, DVC-related, negative cognition; however, from a neurophysiological and psychological level the final, adaptive positive cognition may be of VVC nature. In fact, phase 5 of the EMDR protocol, related to the installation of positive cognition, requires the patients to be asked whether they still feel the initially chosen positive cognition to be true or whether, currently, they would choose another positive cognition. This is not a redundant question since, after the EMDR desensitization-related phase 4, the neurophysiological activation may have shifted toward a more adaptive attitude, hopefully, of VVC nature. However, this is not always the case, and many times, the positive cognition remains of SNS nature at the end of the desensitization-related phase 4. In these cases, we recommend not considering as definitely processed the memories that, at the end of the desensitization-related phase 4, even in the absence of physiological disturbance, still persist with SNS-related positive cognitions.

At the neurophysiological level, it is worth specifying that our suggested mechanisms are not opposed to but may complement other proposed mechanisms (Pagani et al., 2017; Baek et al., 2019). For example, in mice, Baek et al. (2019) identified the SC-MDT-BLA circuit that was involved in the observed persistent fear reduction. SC is believed to be widely involved in eye and body orientation and covert and overt attention (Ignashchenkova et al., 2004; Gandhi and Katnani, 2011), while MDT is believed to relay inputs from the amygdala, monitoring internal movements of the eye, and to play a role in cognitive flexibility, inhibition, fluid intelligence, speed processing, and emotional cognition (Mitchell and Chakraborty, 2013; Li et al., 2022). However, recently SC has been found to be essential for acute dark induction of wakefulness and spontaneous sleep–wake cycle (Zhang et al., 2019), while MDT lesion was found to increase REM sleep (Sriji et al., 2021). Accordingly, the neuronal circuitry identified by Baek et al. (2019) may be involved in sleep-like activity as well. In addition, our proposed mechanisms of action may exert their functions and effectively promote EMDR benefits and traumatic memory reprocessing if perceived safety is taken into account and accurately assessed and promoted in the therapeutic setting. Though the precise neurobiological underpinnings of dissociation remain elusive, there is evidence for a link between dissociative states/traits and altered activity in brain regions involved in emotion processing and memory (e.g., amygdala, hippocampus, parahippocampal gyrus, and middle/superior temporal gyrus), interoception and attention regulation (insula), self-referential processes (e.g., posterior cingulate cortex and precuneus), cognitive control, and arousal modulation (e.g., mPFC and ACC)—functions that may be altered during dissociation (Menon, 2011; van der Kruijs et al., 2014; Lotfinia et al., 2020; Vesuna et al., 2020; Arancibia et al., 2023). These brain regions mostly belong to the default mode network and the salience network (i.e., anterior insula and ACC; Menon and Uddin, 2010; Menon, 2011). At the level of neurocircuitry, COVID-19 patients who may suffer from dissociative symptomatology may benefit from EMDR interventions. ABS of the EMDR may promote the activity of brainstem areas as the NTS, which shows projections to the central nucleus of the amygdala, the ACC, and the mPFC, and promote the reprocessing of autobiographical memories and mitigate dissociative symptomatology. In fact, using fMRI, it was shown that, during the retrieval of an autobiographical event, the BOLD signal in the ventromedial PFC was modulated by the personal significance and emotional intensity of the memory. These results support the idea that the vmPFC processes self-relevant information and suggest that it is involved in representing the personal emotional values of the elements comprising autobiographical memories (Lin et al., 2016). In addition, ACC was related to the ER processing of autobiographical memories (Yang et al., 2022). Since it was shown that traumatic memories are less integrated with other autobiographical information in trauma survivors and this condition fostered dissociative symptoms (Kleim et al., 2008), the EMDR may promote the integration of autobiographical memories and a reduction in dissociative symptomatology.

Though conceived to keep others safe (Brooks et al., 2020), COVID-19 quarantine has exerted an elevated psychological impact on the general population in terms of a lack of perceived safety (Poli et al., 2020). In particular, the quarantine was found to increase the perceived stress level that played a critical role in triggering dissociative experiences among the general population. In addition, the increased frequency of dissociative experiences may promote a higher risk for the emergence of dissociative disorders (Garbóczy et al., 2021). Using neurophysiologically informed psychometric tools to assess perceived safety at treatment entry, and along the treatment, perceived safety can be assessed, promoted, and maintained and, in turn, safety may foster EMDR reprocessing, psychological and neurophysiological integration, and a function as a protective factor from the emergence of dissociative disorders.

## Author contributions

AP: conceptualization and formal analysis. AP and MM: methodology, validation, investigation, resources, data curation, writing—review and editing, and project administration. AP, FC, and JS: writing—original draft preparation. MM: supervision. All authors have read and agreed to the published version of the manuscript.
